# Light Magnesium Oxide Nanosheets for Efficient Fluoride Removal from Geothermal Water: Rapid Adsorption and Mechanistic Insights

**DOI:** 10.3390/molecules31193387

**Published:** 2026-09-23

**Authors:** Junli Chen, Fangjing Xiao, Xiaomei Cui, Duo Bu, Jianjie Fu, Qiangying Zhang

**Affiliations:** 1 Key Laboratory of Biodiversity and Environment on the Qinghai-Tibet Plateau, Ministry of Education, School of Ecology and Environment, Xizang University, Lhasa 850000, China; chenjunli@xzu.edu.cn (J.C.); xfj2023@stu.xzu.edu.cn (F.X.); cuixiaomei@utibet.edu.cn (X.C.); phudor@vip.163.com (D.B.); 2 Analysis and Testing Center, Xizang University, Lhasa 850000, China; 3 State Key Laboratory of Environmental Chemistry and Ecotoxicology, Research Center for Eco-Environmental Sciences, Chinese Academy of Sciences, Beijing 100085, China

**Keywords:** Qinghai–Tibet Plateau, surface reactivity, hydroxyl exchange, adsorption kinetics, adsorption isotherm

## Abstract

Fluoride contamination in geothermal water is a persistent water-quality concern in high-altitude regions, particularly on the Qinghai–Tibet Plateau. In this study, lightweight magnesium oxide (MgO) nanosheets were investigated for fluoride adsorption from geothermal water, with emphasis on adsorption performance and the underlying mechanism. The effects of MgO dosage, temperature, contact time, and initial fluoride concentration were systematically evaluated, while the physicochemical changes of MgO before and after fluoride adsorption were characterized using SEM, EDS, HRTEM, BET, FTIR, and XPS. The MgO nanosheets exhibited abundant surface hydroxyl groups and enabled rapid fluoride uptake under elevated-temperature conditions. At 70 °C and an MgO dosage of 3 g/L, more than 95% of fluoride was adsorbed within 10 min. The saturated adsorption capacity was 87.167 mg/g at an initial fluoride concentration of 500 mg/L. Kinetic analysis revealed rapid initial uptake followed by a slower adsorption stage, with intraparticle diffusion contributing to, but not solely controlling, the overall adsorption process. The equilibrium data were better described by the Langmuir model than by the Freundlich model (R^2^ = 0.940 vs. 0.725), suggesting relatively homogeneous surface sites. FTIR and XPS analyses revealed pronounced changes in the surface chemical environment following water contact and fluoride adsorption. These changes suggest that water-induced hydration and hydroxylation generate reactive Mg–OH sites, which subsequently interact with fluoride through hydroxyl–fluoride exchange and the formation of Mg–F-related surface species. These findings highlight the contribution of surface chemical interactions to fluoride uptake by MgO nanosheets and their potential for rapid fluoride control in geothermal water.

## 1. Introduction

The Qinghai–Tibet Plateau (QTP) is one of the world’s most extensively developed geothermal regions, where prolonged interactions between geothermal fluids and surrounding rocks promote the enrichment of fluoride, arsenic, and boron [1,2,3,4,5]. Among these constituents, fluoride is of particular concern. Previous studies have reported that fluoride concentrations in geothermal waters from representative geothermal areas of the QTP, including Yangbajing, Nagqu, and Cuomei, are generally 2–15 mg/L, with concentrations exceeding 20 mg/L in some samples [6]. These levels substantially exceed the fluoride limit of 1.0 mg/L specified by China’s Standards for Drinking Water Quality (GB 5749-2022) [7]. The discharge of fluoride-rich geothermal waters may further increase fluoride levels in receiving surface waters, posing potential risks to drinking-water safety and aquatic ecosystems [8]. Fluoride is an essential trace element for human health, but its safe intake range is narrow. At appropriate levels, fluoride contributes to the mineralization of teeth and bones and helps prevent dental caries [9]. However, chronic exposure to excessive fluoride can cause dental and skeletal fluorosis and may impair neurological and renal function, whereas insufficient intake may increase the risk of dental caries [10,11,12]. In fluoride-endemic areas of the QTP, particularly those affected by geothermal activity, long-term exposure to fluoride-rich water has been associated with substantial fluorosis burdens. Large-scale epidemiological surveys in Tibet reported dental fluorosis prevalence of 31.81% among children and skeletal fluorosis prevalence of 48.59% among adults [13]. Therefore, effective and sustainable fluoride-removal technologies are important for drinking-water safety and the sustainable utilization of geothermal resources.

Various technologies, including precipitation/coagulation, membrane separation, ion exchange, electrocoagulation, and adsorption, have been developed for fluoride removal [14,15,16,17,18]. However, precipitation/coagulation, membrane separation, ion exchange, and electrocoagulation may be limited by chemical consumption, high energy or capital requirements, membrane fouling, or electrode consumption. Among these technologies, adsorption is particularly attractive because of its operational simplicity, relatively low energy demand, high removal efficiency, and limited generation of secondary pollutants [19]. Its performance, however, strongly depends on the physicochemical properties and surface reactivity of the adsorbent. The performance of fluoride adsorption depends strongly on the physicochemical properties and surface reactivity of the adsorbent. Metal-based adsorbents, including Al-, Fe-, Ti-, Ce-, and Mg-based materials, have been widely investigated for fluoride removal [20,21,22,23,24]. Among them, Mg-based adsorbents are particularly attractive because of their environmental compatibility, availability, and affinity toward fluoride [25]. Mg oxides, Mg hydroxides, and Mg–Al layered double hydroxides have demonstrated effective fluoride uptake through electrostatic attraction, ion exchange, and surface complexation [26]. In particular, MgO nanosheets possess a two-dimensional structure with accessible surface sites and hydroxyl-rich surfaces that may facilitate fluoride uptake through surface interactions and ligand exchange [27,28]. However, their fluoride-removal behavior under the complex hydrochemical conditions of QTP geothermal waters, including alkaline pH, high mineralization, and coexisting ions, remains insufficiently understood [29,30]. Further investigation is therefore needed to clarify their adsorption behavior, kinetics, and underlying mechanisms under these conditions.

Accordingly, this study systematically evaluated the fluoride-removal performance of MgO nanosheets using both simulated fluoride-rich water and actual geothermal water. Structural and surface properties were characterized using complementary techniques, while adsorption kinetic, isotherm, and thermodynamic analyses were conducted to elucidate the adsorption behavior and underlying mechanisms. The results provide insights into the applicability of MgO nanosheets for fluoride-rich geothermal-water treatment and contribute to the development of practical approaches for improving water safety in fluoride-affected geothermal regions.

## 2. Results and Discussion

### 2.1. Structure and Morphology of the Adsorbent

#### 2.1.1. SEM, EDS, HRTEM and SAED Analysis

To elucidate the morphology, elemental composition, and crystal structural evolution of the MgO nanosheets during fluoride adsorption, scanning electron microscopy (SEM), energy-dispersive X-ray spectroscopy (EDS), high-resolution transmission electron microscopy (HRTEM), and selected area electron diffraction (SAED) analyses were performed. As shown in Figure 1a, the pristine MgO exhibited well-defined nanosheet-like morphologies, with lateral dimensions of approximately 200 nm and thicknesses below 100 nm. The nanosheet morphology may facilitate water penetration and promote interfacial contact between the adsorbent and aqueous phase. This structural feature may provide favorable conditions for fluoride diffusion and access to surface-active sites.

The elemental composition and spatial distribution were further examined by EDS mapping. As shown in Figure 1b, the pristine MgO nanosheets were dominated by Mg and O, consistent with the expected composition of MgO. After fluoride adsorption, a distinct F signal appeared and was broadly distributed over the nanosheet surface, together with the Mg and O signals. This emergence of the F signal provides direct elemental evidence for the enrichment of fluoride species on the MgO surface, confirming that fluoride was effectively captured by the adsorbent. The corresponding HRTEM images and EDS spectrum in Figure 1c further illustrate the morphological and compositional changes before adsorption, during hydrolysis, and after fluoride adsorption. Compared with the pristine material, the nanosheet morphology was retained during the hydrolysis and adsorption processes, although localized changes in surface texture and aggregation were observed. Meanwhile, the EDS spectrum of the adsorbed sample clearly contained Mg and O as the major elements, accompanied by a detectable F signal, further supporting fluoride uptake.

The structural evolution of MgO during hydrolysis and fluoride adsorption was further investigated by SAED (Figure 1d). The pristine sample exhibited distinct diffraction rings, indicating the crystalline nature of the MgO nanosheets. During hydrolysis, changes in the diffraction features were observed, suggesting that the original MgO crystal structure underwent partial transformation in the aqueous environment. This observation is consistent with the hydration of MgO to form Mg–OH species, as further supported by the XPS results discussed below. After fluoride adsorption, the diffraction features remained detectable, although their local characteristics differed from those of the pristine sample. These results indicate that the overall crystalline framework was not completely disrupted during adsorption, while localized structural rearrangement or surface transformation may have occurred.

Taken together, the SEM, EDS, HRTEM, and SAED results demonstrate that the MgO nanosheets maintained their overall sheet-like morphology during hydrolysis and fluoride adsorption while undergoing detectable changes in surface composition and local crystal structure. The appearance and distribution of F^−^ after adsorption directly confirmed fluoride enrichment on the adsorbent, whereas the SAED results suggested that MgO underwent partial structural transformation upon contact with water. These observations support a surface-mediated adsorption process involving hydration/hydroxylation of MgO, followed by interactions between F^−^ and the newly formed surface hydroxyl sites. The structural and elemental evolution observed in Figure 1 therefore provides an important basis for further elucidating the fluoride adsorption mechanism through FTIR, XPS, and kinetic analyses.

#### 2.1.2. Porosity Analysis

Nitrogen adsorption–desorption analysis was performed to characterize the textural properties of the MgO nanosheets, with the pore size distribution determined using the Barrett–Joyner–Halenda (BJH) method (Figure 2). The sample exhibited a Brunauer–Emmett–Teller (BET) specific surface area of 3.82 m^2^/g and a Langmuir surface area of 6.74 m^2^/g. The relatively low BET surface area indicates that the material does not possess a highly developed specific surface area. The average pore size was 16.78 nm, with a total pore volume of 0.016 cm^3^/g. These results characterize the surface area and pore properties of the MgO nanosheets. The fluoride adsorption performance was further evaluated based on the surface properties and adsorption experiments.

#### 2.1.3. FTIR Analysis

Fourier-transform infrared spectroscopy (FTIR) was employed to investigate the evolution of the surface chemical environment of the MgO nanosheets before adsorption, during hydrolysis, and after fluoride adsorption, as shown in Figure 3. The pristine MgO nanosheets exhibited a characteristic band at 430.79 cm^−1^, which was assigned to the lattice vibration of Mg–O, confirming the MgO framework [31]. The bands at 3432.62 and 3699.41 cm^−1^ were attributed to O–H stretching vibrations associated with surface hydroxyl groups and adsorbed water, indicating the presence of hydroxylated and hydrated species on the MgO surface [31,32].

During hydrolysis, the Mg–O-related band shifted from 430.79 to 459.63 cm^−1^, accompanied by changes in the band at 1629.74 cm^−1^ and the O–H stretching bands at 3431.79 and 3695.99 cm^−1^ [33]. These spectral changes indicate that contact with water altered the surface chemical environment of MgO, particularly its hydration and hydroxylation states. The shift in the low-wavenumber Mg–O vibration further suggests a change in the local Mg–O coordination environment during hydrolysis, indicating that hydrolysis may contribute to the formation or transformation of reactive surface sites.

Following fluoride adsorption, further changes were observed in the FTIR spectrum. The low-wavenumber band remained near 459.55 cm^−1^, while the band at 1629.75 cm^−1^ remained evident. Meanwhile, the O–H stretching bands at 3641.24 and 3696.51 cm^−1^ exhibited changes relative to the hydrolyzed sample. These variations indicate that fluoride uptake was accompanied by further modification of the hydrated and hydroxylated surface environment of MgO. In particular, the changes in the O–H-related bands suggest that surface hydroxyl groups and interfacial water may participate in fluoride adsorption.

Overall, the FTIR results demonstrate a progressive evolution of the MgO surface from the pristine state through hydrolysis to fluoride adsorption. Hydrolysis modified the local Mg–O coordination environment and the surface hydration/hydroxylation state, potentially promoting the formation or exposure of reactive surface sites. Subsequent fluoride adsorption induced additional changes in the corresponding vibrational features, supporting the involvement of hydrated and hydroxylated surface sites in fluoride uptake. However, FTIR alone is insufficient to unambiguously establish the formation of Mg–F bonds. Therefore, the specific chemical interactions between F^−^ and the MgO surface should be further verified by XPS, particularly through analysis of the F 1s, Mg 1s, and O 1s spectra.

#### 2.1.4. XPS Analysis

To further elucidate the surface chemical evolution of MgO nanosheets during hydrolysis and F^−^ adsorption, X-ray photoelectron spectroscopy (XPS) analyses were performed on the pristine, hydrolyzed, and F^−^-adsorbed samples, as shown in Figure 4. The Mg 1s, O 1s, and C 1s spectra were examined to characterize changes in the surface chemical environment during hydrolysis and subsequent fluoride uptake.

The Mg 1s spectrum of the pristine sample (Figure 4a) exhibited two major components at 1303.98 and 1305.08 eV. The lower-binding-energy component was assigned to Mg–O, whereas the component at approximately 1305 eV was attributed to MgCO_3_ [34]. These results indicate that, in addition to the MgO matrix, carbonate-associated species were present on the sample surface. After hydrolysis (Figure 4b), the relative contributions of the Mg-related components changed, accompanied by an enhanced O–H contribution in the O 1s spectrum. This change supports the hydroxylation of the MgO surface upon contact with water [35]. After F^−^ adsorption (Figure 4c), the Mg 1s spectrum exhibited further changes, with the emergence of a new component at 1304.48 eV. This component was tentatively associated with a Mg–F-related chemical environment [36]. The changes in the Mg 1s and O 1s spectra, together with the O–H-related vibrations observed by FTIR, suggest that F^−^ interacts with the hydroxylated MgO surface, possibly through surface hydroxyl exchange and coordination with reactive Mg sites, resulting in the formation of Mg–F-related species.

Collectively, the XPS and FTIR results support a sequential “hydrolysis–surface hydroxylation–F^−^ interaction” pathway for F^−^ removal by MgO nanosheets. Upon contact with water, the MgO surface undergoes hydration and hydroxylation, as evidenced by the enhanced O–H contribution in the O 1s spectrum. Subsequent interaction with F^−^ induces changes in the surface Mg chemical environment and the emergence of a Mg–F-related component. These findings suggest that F^−^ removal by MgO nanosheets involves surface chemical interactions in addition to physical adsorption, with hydroxyl-mediated interactions and Mg–F coordination potentially contributing to F^−^ uptake.

### 2.2. Optimize the Adsorption Conditions

Adsorbent dosage, temperature, and contact time are important operational parameters governing the adsorption performance of MgO toward F^−^. The effect of adsorbent dosage on the adsorption efficiency of F^−^ is shown in Figure 5a. As the MgO dosage increased from 0.2 to 4.0 g/L, the adsorption efficiency increased from 56.10% to 96.01%, indicating that a higher dosage provided more accessible surface-active sites for F^−^ uptake. Further increasing the MgO dosage resulted in only a marginal improvement in adsorption efficiency, with the overall performance approaching a plateau. Therefore, 3.0 g/L was selected as the optimal adsorbent dosage for subsequent experiments, considering both adsorption efficiency and adsorbent consumption.

Specifically, the reported surface temperature of geothermal water in the Yangbajing geothermal field is approximately 67–80 °C [37]. After discharge, however, geothermal water continuously exchanges heat with the surrounding environment and gradually cools toward ambient temperature. This cooling process can be particularly pronounced under the relatively low ambient temperatures in Xizang. Therefore, adsorption experiments at different temperatures were conducted to assess the effect of this temperature variation on F^−^ removal performance. As shown in the results presented in Figure 5b, within the range of 15–40 °C, both the adsorption efficiency and adsorption capacity increased markedly with increasing temperature. A further increase from 40 to 60 °C resulted in a continued but less pronounced enhancement. When the temperature increased from 60 to 80 °C, the adsorption performance remained relatively stable, indicating that further heating provided limited improvement in F^−^ uptake. It should be emphasized that, for the subsequent adsorption isotherm and kinetic experiments, the geothermal water temperature was not artificially adjusted; instead, the experiments were conducted at 70 °C, representing the actual discharge temperature of the geothermal water.

Contact time is another critical parameter governing adsorption kinetics. As shown in Figure 5c, MgO exhibited rapid F^−^ uptake during the initial stage of adsorption. Within 10 min, the adsorption efficiency exceeded 95%, while the residual F^−^ concentration decreased from approximately 11 mg/L to below 1 mg/L. Further prolongation of the contact time resulted in only a limited change in the residual F^−^ concentration, indicating that MgO could achieve rapid F^−^ uptake within a short contact period. This rapid adsorption behavior may be associated with the readily accessible hydroxylated surface sites of MgO and the rapid interfacial interaction between F^−^ and surface Mg sites. Accordingly, 10 min was selected as the contact time for subsequent experiments, providing a favorable balance between adsorption efficiency and treatment time.

### 2.3. Adsorption Kinetics

The adsorption kinetics of F^−^ onto MgO nanosheets were evaluated using the PFO, PSO, and W–M intraparticle diffusion models (Figure 6), with the corresponding fitting parameters summarized in Table 1. The PFO model provided a better fit to the experimental data than the PSO model, with $R^{2}$ values of 0.973 and 0.847, respectively. The equilibrium adsorption capacity calculated from the PFO model (7.924 mg/g) was also close to the experimentally determined value (7.799 mg/g), whereas the PSO model yielded a calculated value of 8.222 mg/g. These results indicate that the PFO model provides a better empirical description of the overall adsorption kinetics under the tested conditions. However, neither kinetic model alone can determine the underlying adsorption mechanism. To further examine the possible diffusion processes, the W–M intraparticle diffusion model was applied. The $q_{t}$ versus $t^{1/2}$ plot exhibited two distinct linear regions, corresponding to an initial rapid adsorption stage (Stage I) and a subsequent slower diffusion-related stage (Stage II). The $k_{id}$ decreased markedly from 2.831 to 0.085 mg/g/min^1/2^, while the intercept increased from −0.781 to 7.608 mg/g, indicating a substantial decrease in the adsorption rate during the later stage. Importantly, neither fitted line passed through the origin, suggesting that intraparticle diffusion was not the sole rate-controlling step. Overall, the kinetic results indicate that F^−^ adsorption involves multiple sequential processes, with rapid adsorption at readily accessible surface sites followed by a slower diffusion-related stage. These kinetic observations, together with the FTIR and XPS results, support the involvement of surface interactions in F^−^ uptake but do not by themselves establish a specific adsorption mechanism.

### 2.4. Adsorption Isotherms

To further elucidate the equilibrium adsorption behavior of F^−^ onto MgO nanosheets, the experimental data were fitted using the Langmuir and Freundlich isotherm models, as shown in Figure 7, with the corresponding fitting parameters summarized in Table 2. The Langmuir model provided a better fit to the experimental data than the Freundlich model, with $R^{2}$ values of 0.940 and 0.725, respectively. The maximum adsorption capacity calculated from the Langmuir model was 70.817 mg/g, whereas an experimental adsorption capacity of 87.167 mg/g was obtained at an initial F^−^ concentration of 500 mg/L. Although the Langmuir model provided a better empirical description of the equilibrium adsorption data, the fitting result alone does not establish a strictly monolayer adsorption mechanism, particularly considering the relatively low BET specific surface area of the MgO nanosheets. The results instead suggest that the equilibrium adsorption behavior of F^−^ was more consistent with adsorption on relatively homogeneous surface sites. Combined with the FTIR and XPS results, which revealed changes in surface hydroxyl groups and the emergence of Mg–F-related species after F^−^ adsorption, these findings suggest that specific surface active sites and surface chemical interactions contributed to F^−^ uptake.

### 2.5. Adsorption Mechanism

By integrating the morphological, textural, spectroscopic, surface-chemical, and adsorption-modeling results in Figure 1, Figure 2, Figure 3, Figure 4, Figure 5, Figure 6 and Figure 7 and Table 1 and Table 2, the fluoride-removal mechanism of MgO nanosheets was attributed to a coupled process involving mesopore-facilitated mass transfer, surface hydration/hydroxylation, hydroxyl–fluoride exchange, and Mg–F coordination, rather than simple physical adsorption. As shown in Figure 1, the overall sheet-like morphology of MgO was largely preserved after adsorption, while F^−^ was detected and broadly distributed on the adsorbent surface by EDS, confirming effective fluoride uptake. The pH of the geothermal water increased from 8.39 before adsorption to 9.13 after adsorption, corresponding to a ΔpH of 0.74. This increase in pH may be associated with the release of OH^−^ during the interaction between surface Mg–OH groups and F^−^, providing additional evidence for hydroxyl–fluoride exchange during adsorption.

Upon contact with water, MgO underwent hydration and surface hydroxylation. The combined FTIR and XPS results revealed changes in the local structure and surface chemical environment, together with an increased contribution of Mg–OH-related species, indicating water-induced surface activation. This process can be represented as follows:

(1) $$\mathrm{MgO}+H_{2}O\to \mathrm{Mg}-{\mathrm{OH}}_{\left(\mathrm{surface}\right)}$$

The resulting hydroxylated surface provided reactive Mg–OH sites for fluoride capture. After adsorption, changes in the O–H region of the FTIR spectra, together with the decreased contribution of Mg–OH-related species in XPS and the emergence of a Mg–F-related chemical environment, indicated that F^−^ interacted with hydroxylated Mg sites through hydroxyl–fluoride exchange and subsequent Mg–F coordination. The proposed surface reaction can therefore be expressed as follows:

(2) $$\mathrm{Mg}-{\mathrm{OH}}_{\left(\mathrm{surface}\right)}+F^{-}\to \mathrm{Mg}-F_{\left(\mathrm{surface}\right)}+{\mathrm{OH}}^{-}$$

The adsorption kinetics and equilibrium isotherms further supported this mechanistic interpretation. The pseudo-first-order model showed a better fit than the pseudo-second-order model (R^2^ = 0.973 vs. 0.847), indicating that adsorption was not solely controlled by surface reaction kinetics. Weber–Morris analysis revealed two distinct stages (R^2^ = 0.951 and 0.938), corresponding to rapid initial uptake, followed by a slower diffusion or site-occupation process. The Langmuir model also provided a better fit than the Freundlich model (R^2^ = 0.940 vs. 0.725), suggesting preferential adsorption on relatively uniform surface sites. The equilibrium adsorption capacity reached 87.167 mg/g, confirming the high fluoride uptake capacity of the MgO nanosheets. Together with the FTIR and XPS results, these findings support the involvement of both structural accessibility and specific surface interactions in fluoride adsorption.

Overall, fluoride removal by MgO nanosheets can be described as a sequential “hydration–hydroxylation–diffusion–hydroxyl exchange–Mg–F coordination” pathway. In this process, water activates the MgO surface, mesoporous channels facilitate F^−^ transport, and hydroxylated Mg sites subsequently immobilize F^−^ through surface chemical interactions. Thus, the excellent fluoride-removal performance of MgO nanosheets arises from the synergistic contribution of structural accessibility and chemically active Mg–OH/Mg sites, with surface hydroxyl exchange and Mg–F coordination serving as the principal pathways for fluoride immobilization.

### 2.6. Adsorption Performance

To further evaluate the adsorption capacity of MgO nanosheets under different fluoride loading conditions, batch adsorption experiments were conducted to investigate the effects of initial F^−^ concentration on adsorption efficiency and adsorption capacity, as shown in Figure 8. As the initial F^−^ concentration increased, the adsorption capacity progressively increased and approached a plateau, indicating that the higher concentration gradient enhanced the driving force for F^−^ transport toward and occupation of available surface sites. In contrast, the adsorption efficiency gradually decreased with increasing initial F^−^ concentration, which can be attributed to the limited number of accessible adsorption sites on the MgO surface. As the F^−^ loading increased, an increasing fraction of F^−^ remained unbound at equilibrium. MgO exhibited favorable overall adsorption performance over an initial F^−^ concentration range of 10–100 mg/L, with the equilibrium F^−^ concentration maintained below 100 mg/L under the tested conditions.

With a further increase in the initial F^−^ concentration, the adsorption capacity gradually approached a plateau, suggesting progressive saturation of the available surface sites. Under the present experimental conditions, MgO exhibited a static equilibrium adsorption capacity of approximately 87.167 mg/g, demonstrating its substantial F^−^ uptake capacity. This result is consistent with the high adsorption capacity predicted by the Langmuir isotherm and further supports the presence of abundant accessible F^−^-binding sites on the MgO surface. Combined with the FTIR and XPS results, the concentration-dependent adsorption behavior indicates that F^−^ uptake is governed not only by the concentration driving force but also by interfacial interactions involving surface hydroxyl groups and reactive Mg sites. Overall, the high adsorption capacity and rapid adsorption kinetics of MgO nanosheets highlight their potential for the treatment of fluoride-contaminated water.

## 3. Materials and Methods

### 3.1. Experimental Instruments and Reagents

Fluoride concentrations were determined using a fluoride ion-selective electrode (Eutech F090 ION700, Thermo Fisher Scientific, Waltham, MA, USA), and the solution pH was measured and adjusted using a pH meter (PHSJ-6L, Leici, Shanghai, China). A magnetic stirrer (RT2, Cat. No. 88880006, Thermo Fisher Scientific, Waltham, MA, USA) was used to provide continuous agitation and ensure adequate solid–liquid contact during the adsorption experiments. Commercially available lightweight MgO was used as the adsorbent, which was purchased from Tianjin Kermel Chemical Reagent Co., Ltd. (Tianjin, China). Sodium citrate dihydrate (Na_3_C_6_H_5_O_7_·2H_2_O), sodium nitrate (NaNO_3_), and sodium acetate (CH_3_COONa) were purchased from Sinopharm Chemical Reagent Co., Ltd. (Tianjin, China). Sodium fluoride (NaF) was purchased from Zhiyuan Chemical Co., Ltd. (Tianjin, China). Hydrochloric acid (HCl) and perchloric acid (HClO_4_) were obtained from Chengdu Kelong Chemical Co., Ltd. (Chengdu, China). All chemicals were of analytical grade or higher and were used without further purification. Deionized water was used throughout all experiments. Geothermal water samples were collected from the Yangbajing geothermal field, Damxung County, Xizang, China.

### 3.2. Characterization

For comprehensive characterization of the adsorbent before and after fluoride adsorption, SEM (FEI, Lexington, KY, USA), EDS (Oxford Instruments, Abingdon, UK), and HRTEM (FEI, Hillsboro, OR, USA) were employed to examine the surface morphology and elemental distribution. The specific surface area was determined by the BET method, and the pore size distribution was calculated by the BJH method from N_2_ adsorption–desorption isotherms (Micromeritics ASAP 2460, Norcross, GA, USA). Prior to analysis, the samples were degassed at 120 °C for 24 h to remove adsorbed moisture and volatile species. Surface functional groups were characterized by FTIR (Shimadzu, Kyoto, Japan) over a wavenumber range of 4000–400 cm^−1^. XPS (Thermo Fisher Scientific, Waltham, MA, USA) was used to determine the elemental composition and surface chemical states, with the C 1s peak used as the internal reference for binding-energy calibration.

### 3.3. Experimental Procedures

#### 3.3.1. Determination of Fluoride Ions by Ion-Selective Electrode Method

Fluoride concentrations were determined using a fluoride ion-selective electrode, and the fluoride-removal efficiency was subsequently calculated. A series of fluoride standard solutions with concentrations of 0.5, 1, 2, 5, 10, and 20 mg/L were prepared. For each standard solution, 10 mL was transferred into a test vessel and mixed thoroughly with 1 mL of total ionic strength adjustment buffer (TISAB). The fluoride ion-selective electrode was then immersed in the solution, and the corresponding electrode potential (*E*) was recorded. A calibration curve was constructed by plotting the measured *E* against the logarithm of the fluoride concentration (log *C*). The resulting linear relationship was expressed as *E* = −*k*(log *C*) + b(−58.412X + 104.16), with a slope of −58.412 mV per decade and a coefficient of determination (*R*^2^) of 0.9997, indicating excellent linearity over the investigated concentration range.

#### 3.3.2. Batch Adsorption Experiments

Batch adsorption experiments were conducted to systematically evaluate the fluoride-removal performance of MgO nanosheets and to investigate the effects of adsorbent dosage, temperature, and contact time. Unless otherwise specified, a known mass of MgO nanosheets was added to geothermal water at a predetermined solid-to-liquid ratio, and the suspension was continuously agitated at 150 rpm in a thermostatic shaking incubator or water bath. After the designated reaction time, the suspension was filtered through a 0.22 μm membrane filter, and the residual fluoride concentration in the filtrate was determined using the fluoride ion-selective electrode method; all residuals were determined in triplicate.

To determine the optimal MgO dosage, different amounts of MgO nanosheets were added to 30 mL of geothermal water to obtain solid-to-liquid ratios of 0.2, 0.5, 1, 2, 3, and 4 g/L. The suspensions were incubated at 80 °C and agitated at 150 rpm until adsorption equilibrium was reached. The suspension was then filtered through a 0.22 μm membrane filter, and the residual fluoride concentration was determined.

Temperature-dependent adsorption experiments were performed at 15, 30, 40, 50, 60, 70, and 80 °C. The MgO dosage was fixed at a solid-to-liquid ratio of 2 g/L, and the suspensions were continuously agitated at 150 rpm for 240 min at the designated temperatures. After treatment, the suspensions were filtered through 0.22 μm membrane filters, and the fluoride concentration in the filtrates was determined.

To investigate the time-dependent fluoride-removal behavior and determine the appropriate contact time, 1.0 g of MgO nanosheets was added to 500 mL of geothermal water, corresponding to a solid-to-liquid ratio of 2 g/L. The suspensions were maintained at 70 °C and agitated at 150 rpm. Samples were collected at predetermined contact times of 2, 5, 10, 20, 30, 60, 120, and 240 min. At each sampling time, an aliquot was immediately withdrawn and filtered through a 0.22 μm membrane filter, followed by determination of the residual fluoride concentration.

Adsorption kinetic models describe the temporal evolution of the adsorption capacity and can provide insights into the rate-controlling processes and interactions governing adsorbate uptake [38]. To further elucidate the adsorption behavior of fluoride onto MgO nanosheets, the PFO, PSO, and W–M kinetic models were fitted to the experimental data using Equations (3)–(5), and the resulting kinetic parameters were used in conjunction with the experimental observations to assess the rate-controlling processes and infer the underlying adsorption mechanism.

The pseudo-first-order (PFO) kinetic model was expressed as

(3) $$q_{t}=q_{e}\left(1-e^{-k_{1}t}\right)$$

The pseudo-second-order (PSO) kinetic model was expressed as

(4) $$q_{t}=\frac{q_{e}^{2}k_{2}t}{1+q_{e}k_{2}t}$$

Weber–Morris (W–M) intraparticle diffusion model was expressed as

(5) $$q_{t}=k_{id}t^{1/2}+C$$

where $q_{e}$ (mg/g) represents the equilibrium adsorption capacity; $q_{t}$ (mg/g) denotes the adsorption capacity at time t; $k_{1}$ (min^−1^) is the pseudo-first-order rate constant; $k_{2}$ (g/mg/min) is the pseudo-second-order rate constant; $k_{id}$ (mg/g/min^1/2^) is the intraparticle diffusion rate constant; C (mg/g) is the intercept, which reflects the thickness of the boundary layer or the contribution of external mass-transfer resistance; and t (min) is the adsorption contact time.

The Langmuir and Freundlich adsorption isotherm models were employed to further evaluate the equilibrium adsorption behavior of fluoride onto MgO nanosheets [39]. These two models were used to characterize the equilibrium adsorption behavior of fluoride and to elucidate the surface characteristics of the MgO nanosheets. The Langmuir and Freundlich models are described by Equations (6) and (7), respectively.

The Langmuir adsorption isotherm model was expressed as

(6) $$q_{e}=\frac{q_{m}k_{L}c_{e}}{1+k_{L}c_{e}}$$

The Freundlich adsorption isotherm model was expressed as

(7) $$q_{e}=k_{F}{c_{e}}^{1}{/}_{n}$$

where $c_{e}$ (mg/L) represents the equilibrium fluoride concentration in solution, $q_{m}$ (mg/g) is the maximum monolayer adsorption capacity, $k_{L}$ (L/mg) is the Langmuir adsorption equilibrium constant, $k_{F}$ (L/mg) is the Freundlich adsorption capacity constant, and n is the Freundlich heterogeneity parameter.

## 4. Conclusions

This study evaluated the fluoride adsorption performance and mechanism of lightweight MgO nanosheets. Under the tested conditions, the nanosheets achieved over 95% adsorption efficiency within 10 min at 70 °C, with an experimental equilibrium adsorption capacity of 87.167 mg/g at an initial F^−^ concentration of 500 mg/L. Increasing the initial F^−^ concentration increased the adsorption capacity but decreased the adsorption efficiency, indicating the influence of the available adsorption sites. Kinetic analysis showed rapid fluoride uptake followed by a slower adsorption stage, and the intraparticle diffusion plots did not pass through the origin, suggesting that intraparticle diffusion was not the only rate-controlling process. The Langmuir model provided a better fit to the equilibrium data than the Freundlich model. FTIR and XPS analyses revealed changes in surface hydration and hydroxylation after water contact, followed by changes associated with fluoride interactions with surface hydroxylated Mg sites. These findings suggest that the resulting hydroxylated surface provides active sites for subsequent F^−^ uptake. Overall, MgO nanosheets exhibited rapid fluoride adsorption and substantial adsorption capacity under the tested conditions, demonstrating their potential for treating fluoride-rich geothermal water.

## Figures and Tables

**Figure 1:**
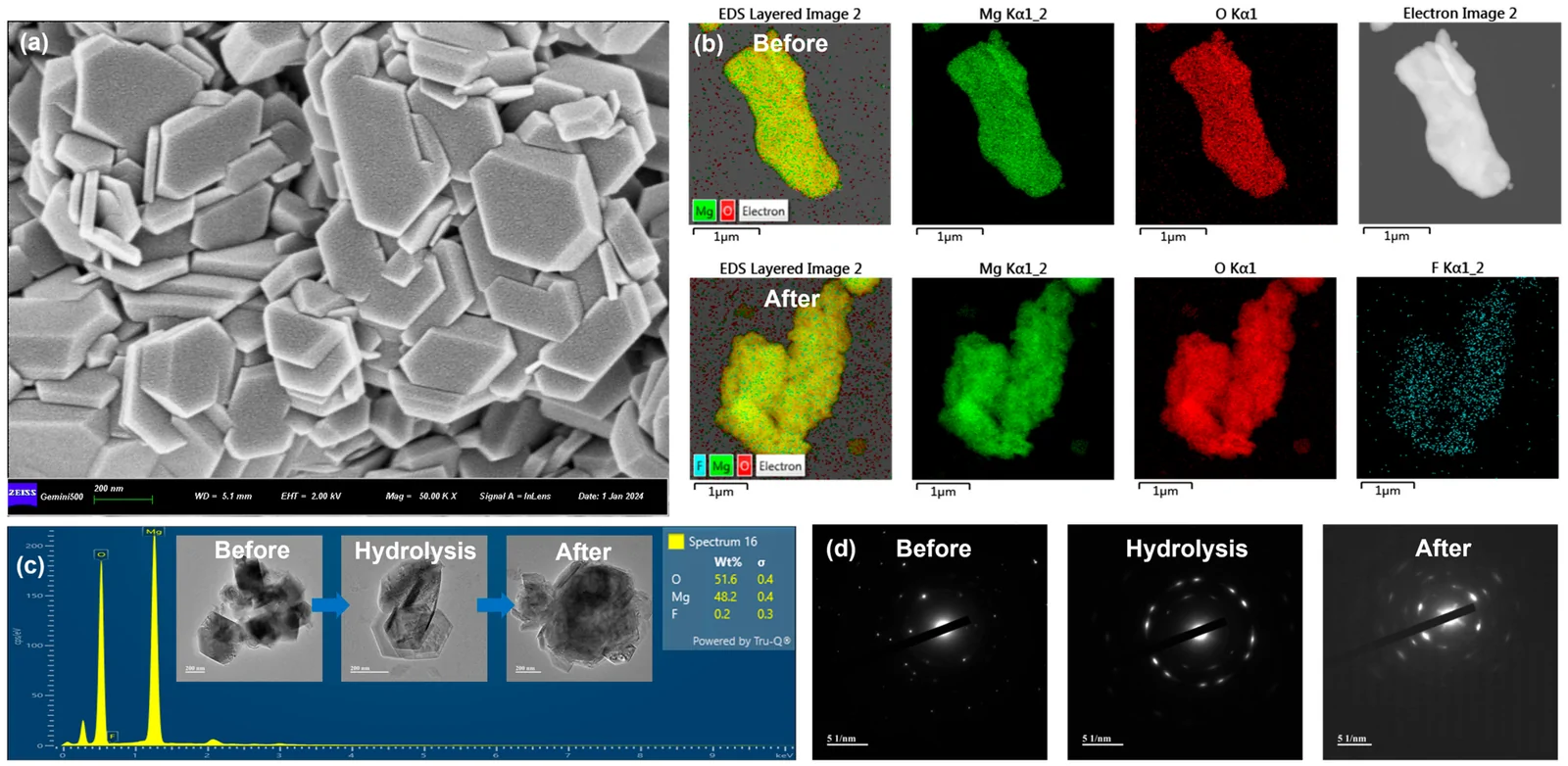
Morphological, elemental, and crystal structural characterization of MgO nanosheets before adsorption, during hydrolysis, and after fluoride adsorption: (**a**) SEM image; (**b**) EDS elemental mapping; (**c**) HRTEM images and EDS spectra; (**d**) SAED patterns.

**Figure 2:**
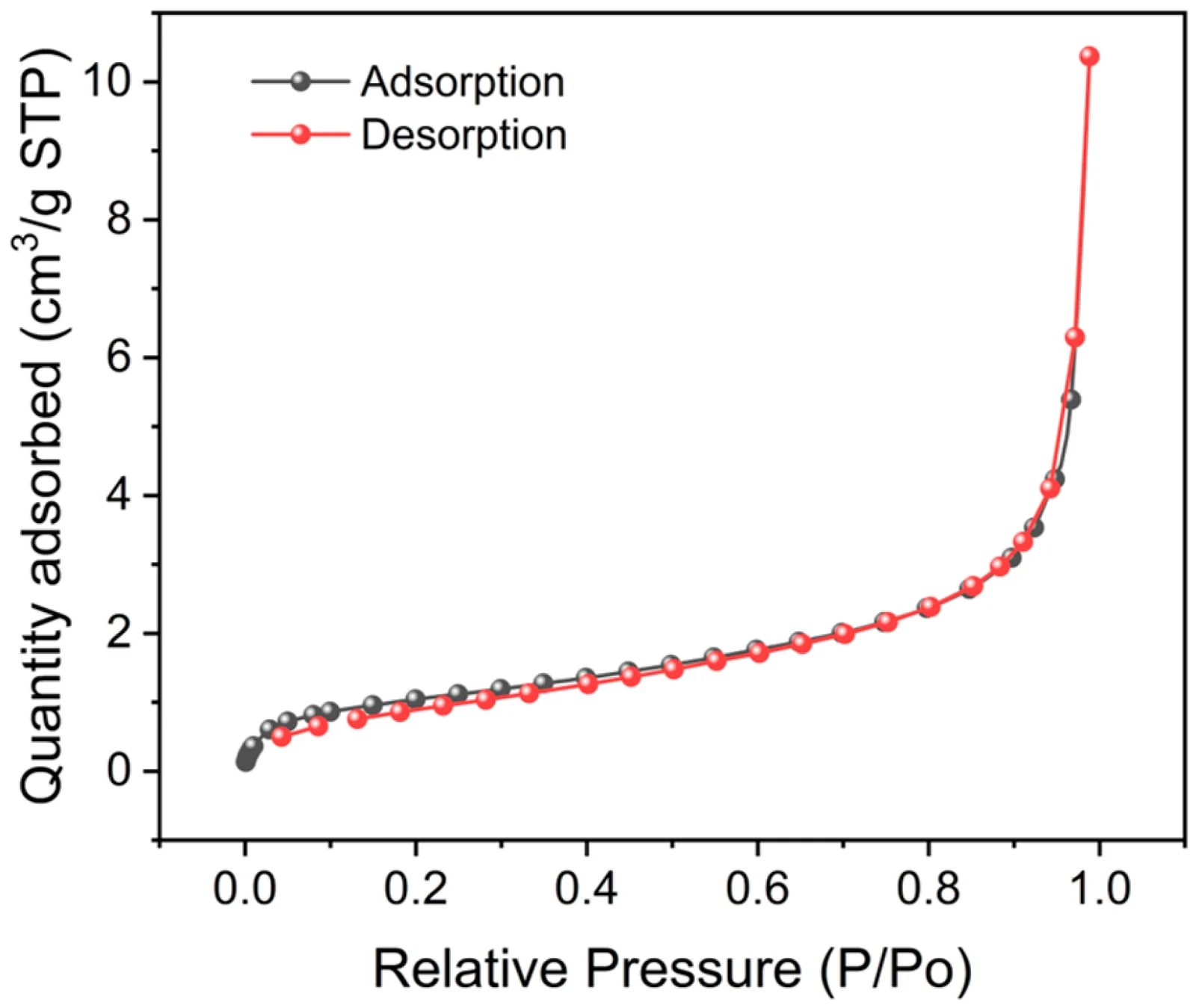
N_2_ adsorption–desorption isotherm of MgO.

**Figure 3:**
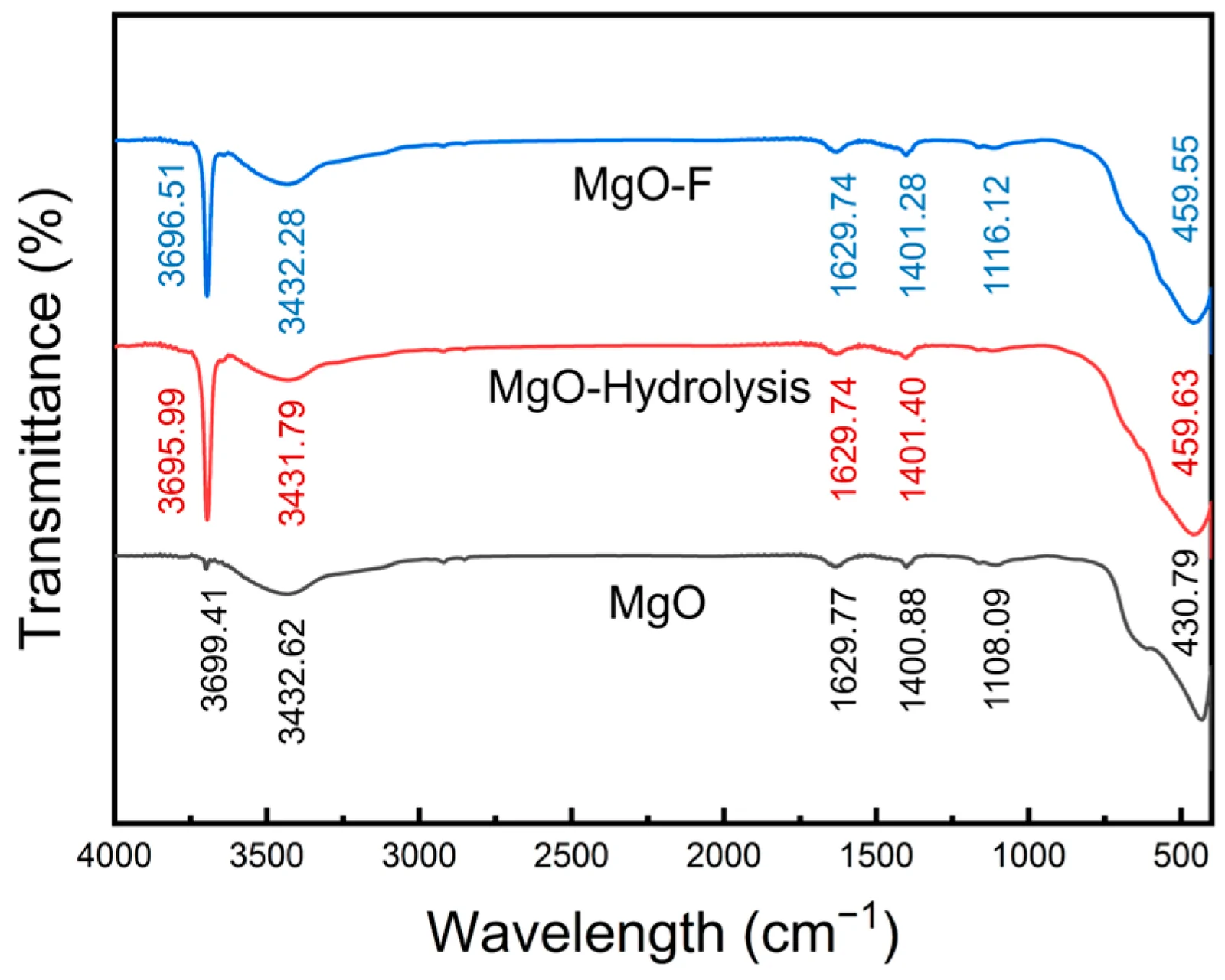
FTIR spectra of MgO before adsorption, during hydrolysis, and after fluoride adsorption.

**Figure 4:**
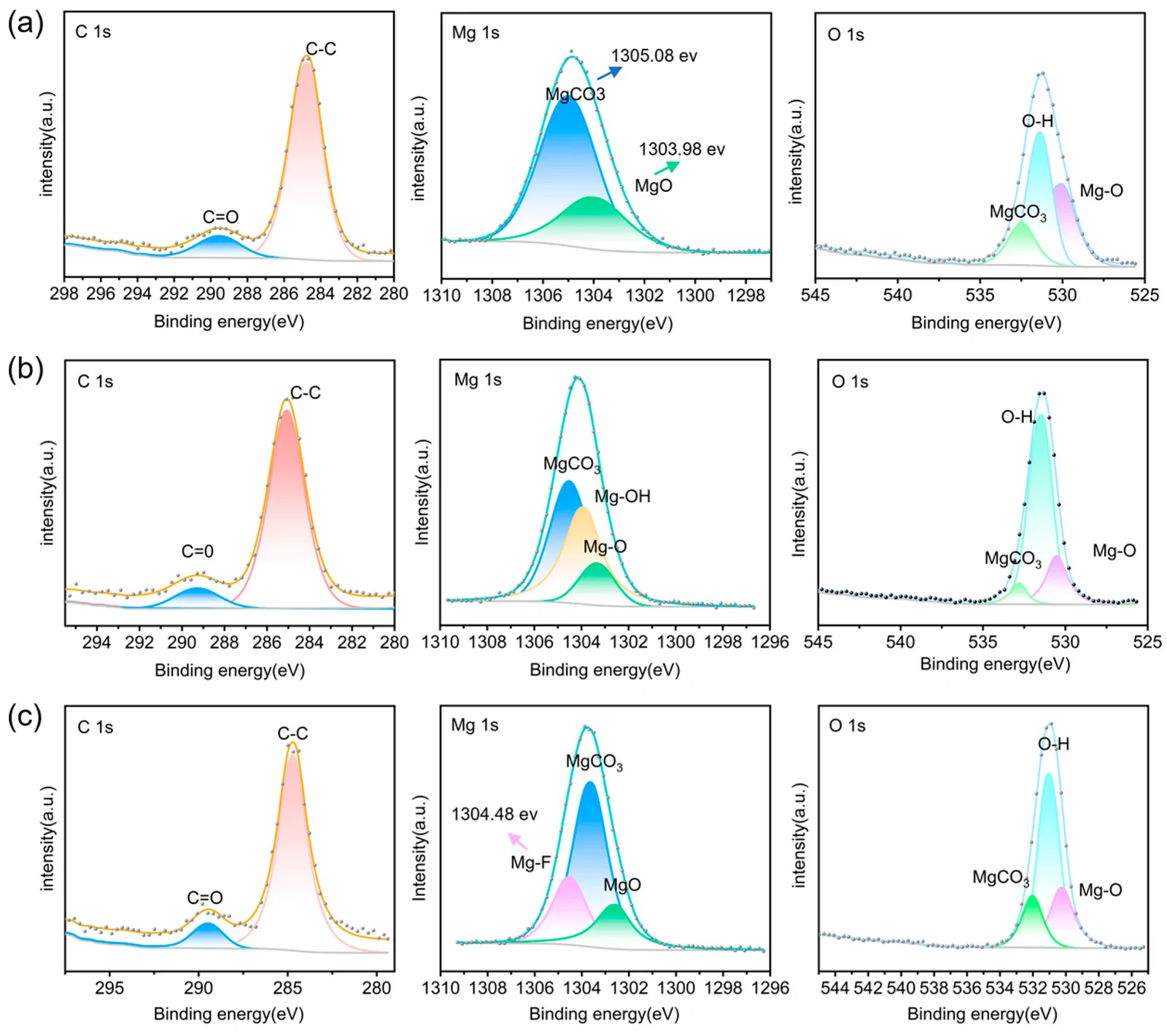
XPS spectra of MgO nanosheets (**a**) before adsorption, (**b**) after hydrolysis, and (**c**) after fluoride adsorption.

**Figure 5:**
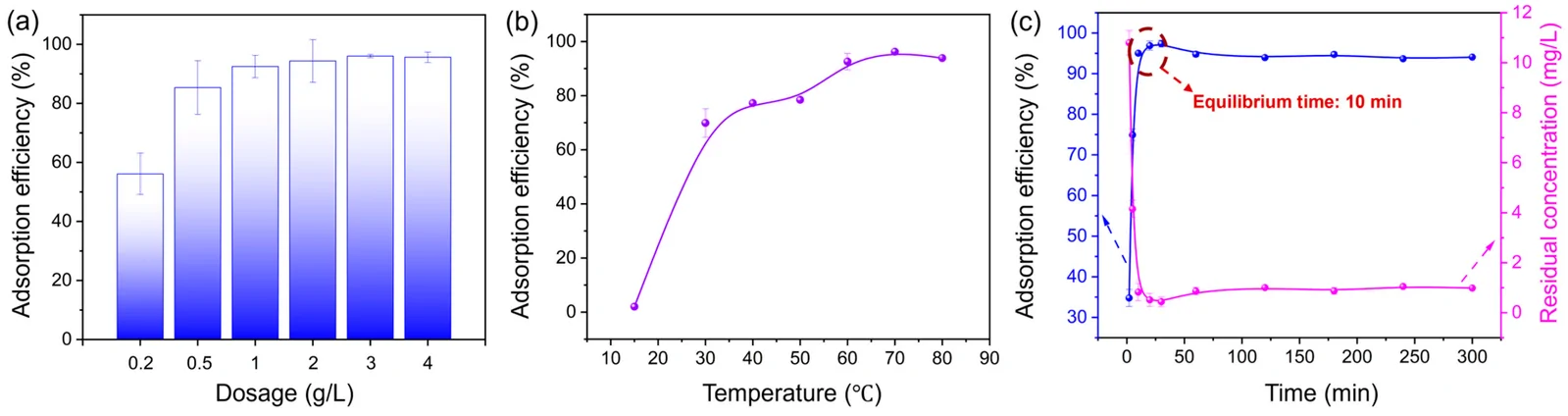
Optimization of adsorption conditions: (**a**) adsorbent dosage; (**b**) temperature; (**c**) time.

**Figure 6:**
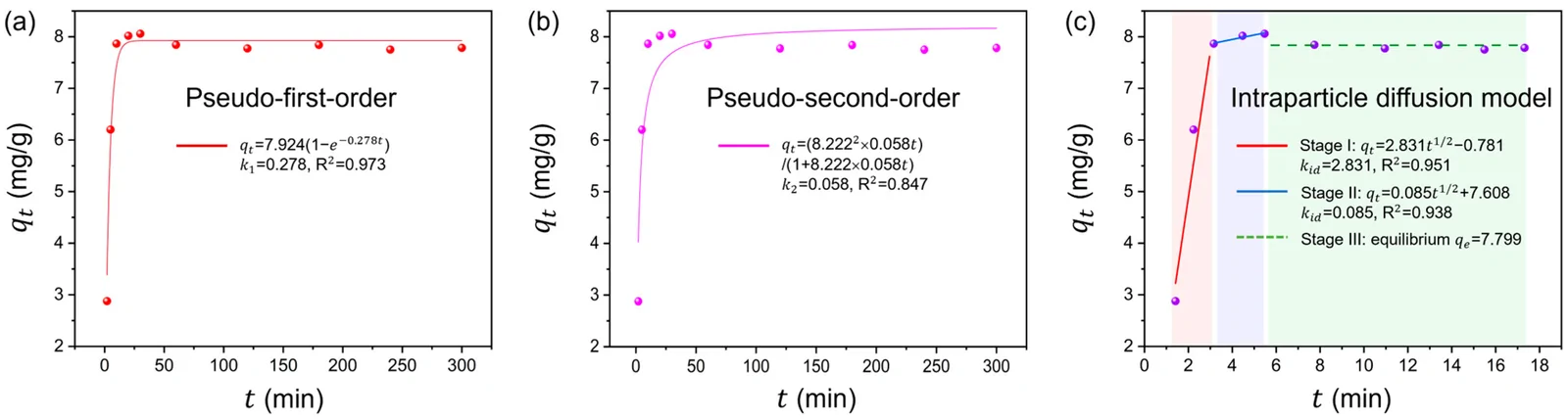
Adsorption kinetic plots based on (**a**) the pseudo-first-order, (**b**) pseudo-second-order, and (**c**) Weber–Morris intraparticle diffusion models.

**Figure 7:**
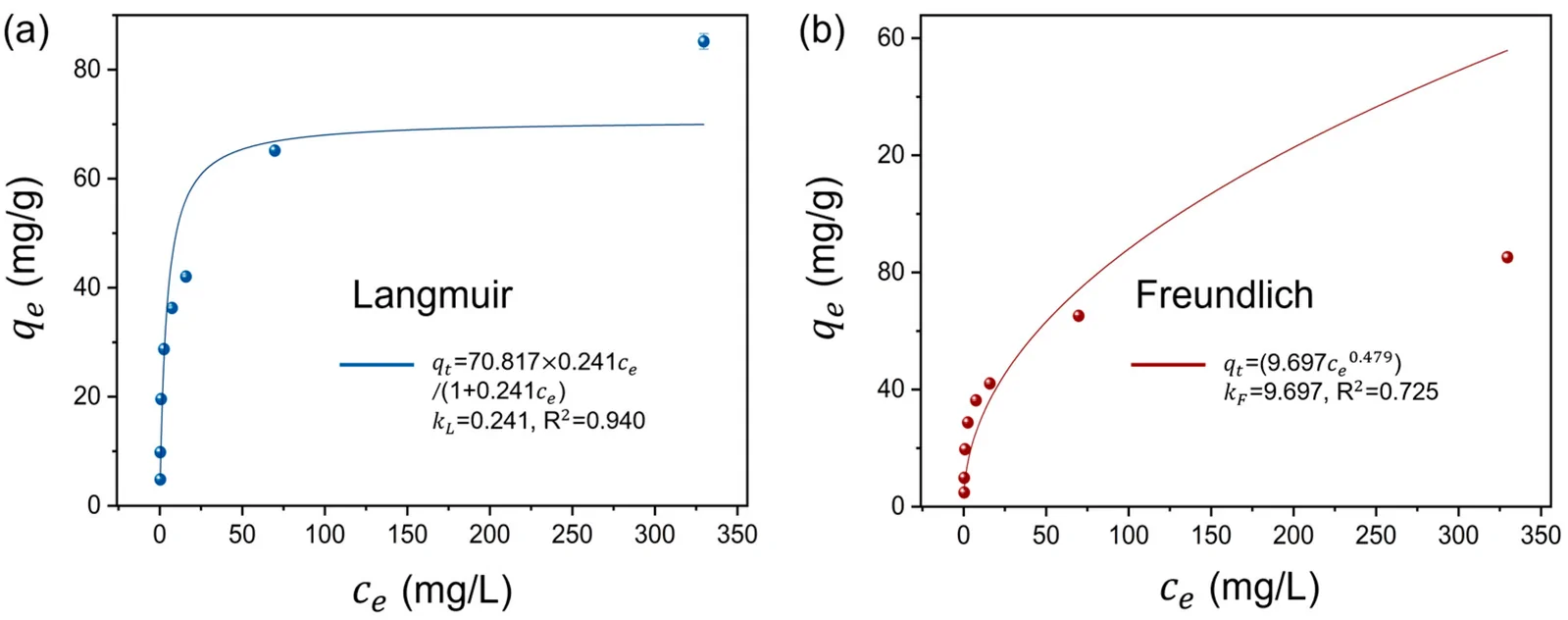
Adsorption isotherm fitting using (**a**) the Langmuir and (**b**) Freundlich models.

**Figure 8:**
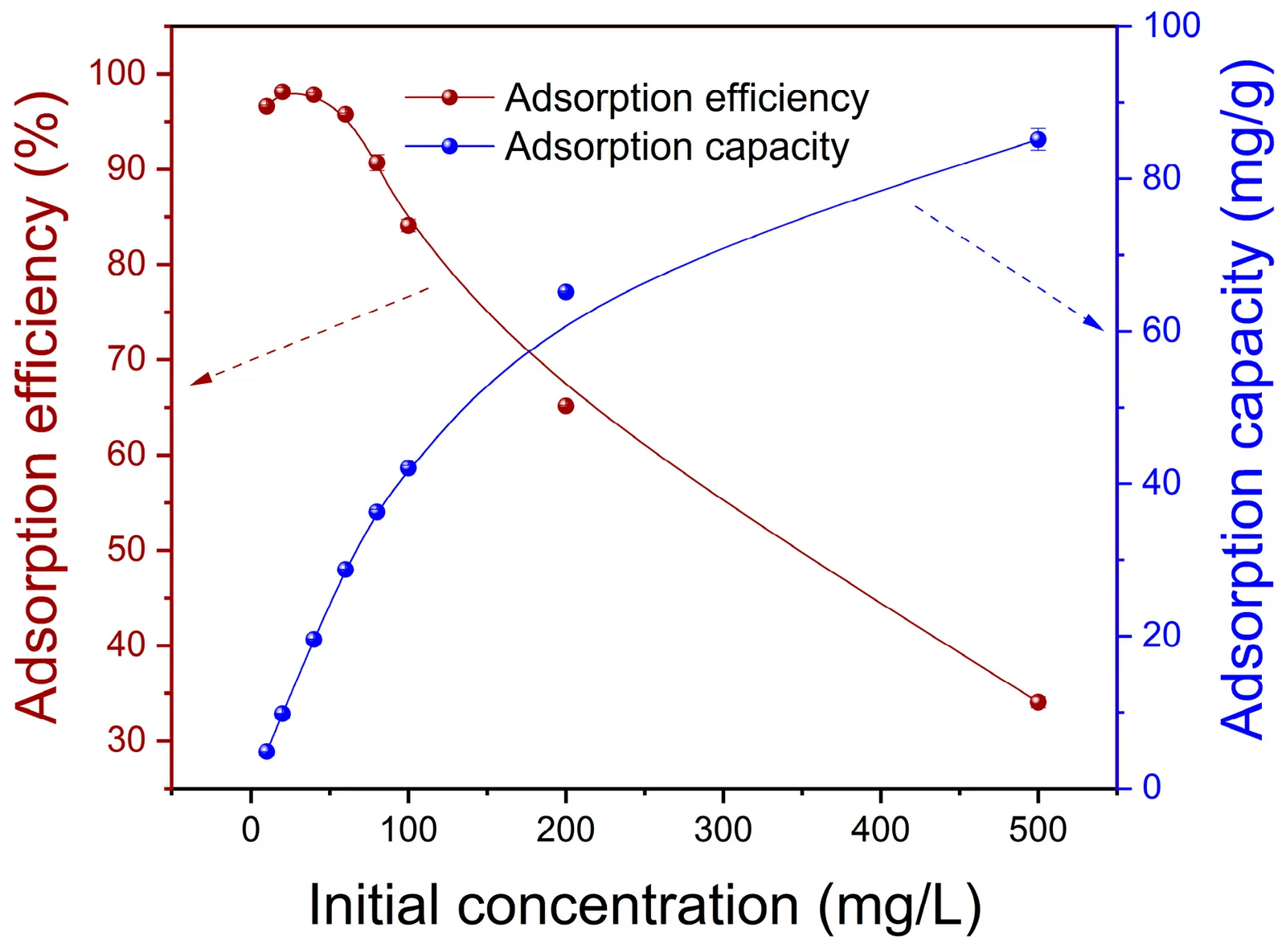
Effects of initial fluoride concentration on adsorption efficiency and adsorption capacity.

**Table 1:** Adsorption kinetic fitting parameters.

Pseudo-First-Order				Pseudo-Second-Order			Intraparticle Diffusion Model					
q_e_ ^1^ (mg/g)	q_cal_ ^2^ (mg/g)	k_1_ (min^−1^)	R^2^	q_cal_ (mg/g)	k_2_ (g/mg/min)	R^2^	Stage I			Stage II		
							k_id_ (mg/g/min^1/2^)	C (mg/g)	R^2^	k_id_ (mg/g/min^1/2^)	C (mg/g)	R^2^
7.799	7.924	0.278	0.973	8.222	0.058	0.847	2.831	−0.781	0.951	0.085	7.608	0.938

^1^ q_e_: experimentally determined equilibrium adsorption capacity (mg/g); ^2^ q_cal_: equilibrium adsorption capacity calculated from the kinetic model (mg/g).

**Table 2:** Adsorption isotherms’ fitting parameters.

Langmuir				Freundlich		
q_e_ ^1^ (mg/g)	q_m_ ^2^ (mg/g)	*k_L_* (L/mg)	R^2^	*k_F_* (mg/g)(L/mg)^1/n^	1/n	R^2^
87.167	70.817	0.241	0.940	9.697	0.479	0.725

^1^ q_e_: experimentally determined equilibrium adsorption capacity (mg/g); ^2^ q_m_: maximum adsorption capacity calculated from the Langmuir isotherm model (mg/g).

## Data Availability

The data presented in this study are available upon reasonable request from the corresponding author.

## References

[1] Le Pape F., Jones A., Vozar J., Wei W. (2012). Penetration of crustal melt beyond the Kunlun Fault into northern Tibet. Nat. Geosci..

[2] Zhang Y., Yuan X., Yang C., Zhong Y., Zhou P., Liu G., Wang Y., Wu H. (2026). Genesis of multi-source mixed geothermal fluids in the eastern Tibetan Plateau. Geosci. Front..

[3] Huang X., Wang S., Wang S., Xiao Z., Zhang M., Zhang H., Qi S. (2025). Fluoride in geothermal water: Occurrence, origin, migration and environmental impact. J. Geochem. Explor..

[4] Shi S., Feng Q., Zhang H., Zhao W., Zhou J., Zhu G., Niu Z., Zhang S. (2024). Arsenic in the Tibetan Plateau’s geothermal systems: A detailed analysis of forms, sources, and geochemical behaviors. Discov. Appl. Sci..

[5] Elenga H.I., Tan H., Su J., Yang J. (2021). Origin of the enrichment of B and alkali metal elements in the geothermal water in the Tibetan Plateau: Evidence from B and Sr isotopes. Geochemistry.

[6] Zhang Q., Tan X., Yu T. (2023). Effectively arsenic (V) and fluoride removal in geothermal water using magnetic Fe_3_O_4_@MgO nanoparticles. Chin. Chem. Lett..

[7] Xu G., Xiao F., Cui X., Bu D., Zhang Q. (2024). Concentrations and sources of arsenic and fluoride in high-temperature geothermal water from Yangbajing, Xizang. Rock Miner. Anal..

[8] Yang Y., Zhang R., Deji Y., Li Y. (2024). Hotspot mapping and risk prediction of fluoride in natural waters across the Tibetan Plateau. J. Hazard. Mater..

[9] Taher M.K., Momoli F., Go J., Hagiwara S., Ramoju S., Hu X., Jensen N., Terrell R., Hemmerich A., Krewski D. (2024). Systematic review of epidemiological and toxicological evidence on health effects of fluoride in drinking water. Crit. Rev. Toxicol..

[10] Zhang Y., Gao Y., Liu X. (2024). Focus on cognitive impairment induced by excessive fluoride: An update review. Neuroscience.

[11] Lavanya S., Shree K.H., Ramani P. (2024). Fluoride effect on renal and hepatic functions: A comprehensive decade review of in vitro and in vivo studies. J. Oral Biol. Craniofac. Res..

[12] Chang R., Zhao W., Zhou B., Ommati M.M., Wang H. (2025). Environmental fluoride exposure and bone metabolism: Molecular pathways and health implications. J. Environ. Sci..

[13] Cao J., Zhao Y., Liu J. (2000). Fluoride in the environment and brick-tea-type fluorosis in Tibet. J. Fluor. Chem..

[14] Pardey A., Bhowal A., Das P., Karmakar S. (2026). Fluoride removal by precipitation-coagulation in high gravity reactors. Chem. Eng. Process. Process Intensif..

[15] Gao X., Liu J., Gai N., Wang J., Yuan Z., Zhou B., Hou R., Yuan R., Chen H. (2025). Induced crystallization techniques for fluoride removal from wastewater: Current developments and applications. J. Environ. Chem. Eng..

[16] Rathi B.S., Kumar P.S., Rangasamy G., Badawi M., Aminabhavi T.M. (2024). Membrane-based removal of fluoride from groundwater. Chem. Eng. J..

[17] Du C., Li Z. (2024). Bibliometric analysis and systematic review of fluoride-containing wastewater treatment: Development, hotspots and future perspectives. J. Environ. Manag..

[18] Nguyen K.A., Shin Y.U., Nguyen D.A., Jang A. (2025). A robust approach for sustainable removal and recovery of fluoride via cryolite crystallization in an enhanced electrocoagulation process. J. Water Process Eng..

[19] Xiao F., Jiang G., Bu D., Fu J., Cui X., Zhang Q. (2025). Rapid and synergistic adsorption of arsenic and fluoride by bifunctional Al/Ce oxides in complex geothermal matrices. Ecotoxicol. Environ. Saf..

[20] Zhao Z., Du X., Wang S., Ling C., Song Y., Chen N., Sun K., Tian L., Zhang Q. (2026). Unlocking internal aluminum reactivity in nanohydrated alumina via amyloid fibril nanoconfinement for highly efficient water defluorination. Chem. Eng. J..

[21] Jeyaseelan A., Naushad M., Ahamad T., Viswanathan N. (2021). Design and development of amine functionalized iron based metal organic frameworks for selective fluoride removal from water environment. J. Environ. Chem. Eng..

[22] Colledge G.T., Outram J.G., Millar G.J. (2022). Improved remediation of fluoride contaminated water using titania-alumina sorbents. J. Water Process Eng..

[23] Lu Y., Wei W., Liu S., Zhang X., Tu Y., Li Y., Peng J., Huang R., Huang X. (2026). Efficient fluoride removal by Ce-MOFs and Ce/Mg MOFs: A mechanistic investigation. ACS Omega.

[24] Kitemangu A.M., Machunda R.L., Rwiza M.J., Malima N.M., Banyikwa A.T., Mataba G.R. (2026). Enhanced fixed-bed column adsorption using a ternary MgO-impregnated eggshell-kaolin composite: Toward an efficient and sustainable fluoride removal technology. Adsorpt. Sci. Technol..

[25] Chan Y.R., Lin W.H., Lin W.Z., Surampalli R.Y., Yu Y.L., Kao C.M. (2025). Fluoride removal by MgO-based adsorbents synthesized via distinct routes: Comparative evaluation of adsorption efficiency and column-scale stability. J. Water Process Eng..

[26] Li Q., Jin Z., Zhou J., Zhou S. (2026). A novel composite of Mg-Al layered double hydroxide/aeolian sand for enhanced removal of fluoride from water. Environ. Geochem. Health.

[27] Wang D.C., Xu M.D., Jin Z., Xiao Y.F., Chao Y., Li J., Chen S., Ding Y. (2023). Synthesis and characterization of porous MgO nanosheet-modified activated carbon fiber felt for fluoride adsorption. Nanomaterials.

[28] Yu M., Gan Z., Liao T., Tang A., Yang H. (2025). Interfacial hydration of zeolite-confined MgO for enhanced fluoride removal from water. Chem. Eng. J..

[29] Song M., Lang X., Zhong Z., Kang F., Nan D., Li H., Yu H., Liu S., Han S., Liu Z. (2022). Hydrogeochemical characteristics and genesis of the Chazi geothermal field area in Tibet. Geofluids.

[30] Adam F.A., Ghoniem M.G., Diawara M., Rahali S., Abdulkhair B.Y., Elamin M.R., Aissa M.A.B., Seydou M. (2022). Enhanced adsorptive removal of indigo carmine dye by bismuth oxide doped MgO based adsorbents from aqueous solution: Equilibrium, kinetic and computational studies. RSC Adv..

[31] Digne M., Sautet P., Raybaud P., Euzen P., Toulhoat H. (2002). Hydroxyl groups on γ-alumina surfaces: A DFT study. J. Catal..

[32] Kurosawa R., Takeuchi M., Ryu J. (2021). Fourier-transform infrared and X-ray diffraction analyses of the hydration reaction of pure magnesium oxide and chemically modified magnesium oxide. RSC Adv..

[33] Singh J.P., Kim S.H., Kang H.K., Won S.O., Lee I.J., Chae K.H. (2018). Are organic templates responsible for the optical and magnetic response of MgO nanoparticles?. Mater. Chem. Front..

[34] Ding Z., Yuan Q., Wang H., Tang Y., Tan Y., He Q. (2022). Anticorrosion behaviour and tribological properties of AZ31 magnesium alloy coated with Nb_2_O_5_/Nb_2_O_5_–Mg/Mg layer by magnetron sputtering. RSC Adv..

[35] Wu S., Wang S., Shao Z., Wang Y., Xiong P. (2025). Self-powered near-infrared mechanoluminescence through MgO/MgF_2_ piezo-photonic heterojunctions. Nat. Commun..

[36] Li J., Fan X., Chen Y., Xiong H., Yu X., Xu M., Zhou Y. (2026). Hydrochemical characteristics of high-temperature geothermal fluids and quantitative assessment of wellbore scaling in the Yangbajain geothermal field, Tibet. Geofluids.

[37] Yang H., Yuan X., Chen Y., Liu J., Zhan C., Lv G., Hu J., Sun M., Zhang Y. (2023). Geochemical evidence constraining genesis and mineral scaling of the Yangbajing geothermal field, Southwestern China. Water.

[38] Vareda J.P. (2023). On validity, physical meaning, mechanism insights and regression of adsorption kinetic models. J. Mol. Liq..

[39] Danat B.T., Wuana R.A., Chahul H.F., Iorungwa M.S. (2026). Review of adsorption isotherms models. Appl. Water Sci..

